# Peroxisomal Stress Response and Inter-Organelle Communication in Cellular Homeostasis and Aging

**DOI:** 10.3390/antiox11020192

**Published:** 2022-01-19

**Authors:** Jinoh Kim, Hua Bai

**Affiliations:** Department of Genetics, Development and Cell Biology, Iowa State University, Ames, IA 50011, USA

**Keywords:** peroxisome, reactive oxygen species, acetyl-CoA, plasmalogen, ER stress, mitochondrial dysfunction, apoptosis, pexophagy

## Abstract

Peroxisomes are key regulators of cellular and metabolic homeostasis. These organelles play important roles in redox metabolism, the oxidation of very-long-chain fatty acids (VLCFAs), and the biosynthesis of ether phospholipids. Given the essential role of peroxisomes in cellular homeostasis, peroxisomal dysfunction has been linked to various pathological conditions, tissue functional decline, and aging. In the past few decades, a variety of cellular signaling and metabolic changes have been reported to be associated with defective peroxisomes, suggesting that many cellular processes and functions depend on peroxisomes. Peroxisomes communicate with other subcellular organelles, such as the nucleus, mitochondria, endoplasmic reticulum (ER), and lysosomes. These inter-organelle communications are highly linked to the key mechanisms by which cells surveil defective peroxisomes and mount adaptive responses to protect them from damages. In this review, we highlight the major cellular changes that accompany peroxisomal dysfunction and peroxisomal inter-organelle communication through membrane contact sites, metabolic signaling, and retrograde signaling. We also discuss the age-related decline of peroxisomal protein import and its role in animal aging and age-related diseases. Unlike other organelle stress response pathways, such as the unfolded protein response (UPR) in the ER and mitochondria, the cellular signaling pathways that mediate stress responses to malfunctioning peroxisomes have not been systematically studied and investigated. Here, we coin these signaling pathways as “peroxisomal stress response pathways”. Understanding peroxisomal stress response pathways and how peroxisomes communicate with other organelles are important and emerging areas of peroxisome research.

## 1. Introduction

Eukaryotic cells contain a variety of membrane-enclosed organelles. Among them, peroxisomes are ubiquitous single membrane-bound organelles that play essential roles in cellular metabolism. Peroxisomes are the major site for both the production and degradation of hydrogen peroxide (H_2_O_2_) due to their high content of peroxisomal oxidases and antioxidant enzymes, such as catalase. Peroxisomes are also critical for the biosynthesis of ether phospholipids, cholesterol, bile acids, and polyunsaturated fatty acids (PUFAs). The importance of peroxisomes is evidenced by peroxisomal biogenesis disorders (PBDs), rare genetic diseases caused by mutations in any of the 13 different *PEX* genes encoding peroxins responsible for the import of membrane or matrix proteins to peroxisomes [1]. Zellweger syndrome (ZS) is the most severe PBD, and patients with ZS show severe brain, liver, kidney, and bone malfunctions [2].

Given the essential roles of peroxisomes in cellular and metabolic homeostasis [3,4], it is not surprising to find that peroxisomal dysfunction is strongly linked to the loss of redox homeostasis, dysregulated lipid metabolism, mitochondrial dysfunction, altered gene expression, and elevated ER stress and cell death. More recently, it has become clear that peroxisome deficiency can also alter functions of specific subcellular organelles [5,6,7], such as mitochondria, endoplasmic reticulum (ER), and lysosome. Peroxisomal stress signals can also be transduced from peroxisomes to the nucleus, as peroxisomal dysfunction often induces global transcriptional changes [8,9]. Thus, similar to other organelle stress pathways (e.g., ER stress), there are specific peroxisomal stress response pathways that allow eukaryotic cells to cope with malfunctioning peroxisomes [10].

Peroxisomes can communicate with other organelles through membrane contact sites, peroxisome-derived metabolites, as well as retrograde signaling. However, it remains largely unclear how peroxisomal stress response pathways are activated and how they protect cells from damages. Understanding peroxisomal stress response pathways and how peroxisomes communicate with other organelles are important areas of peroxisome research because of their vital role in cellular homeostasis, aging, and aging-related diseases [11,12,13,14]. In this review, we first describe peroxisomal biogenesis, the peroxisome protein import machinery, and peroxisome metabolic functions. Next, we summarize recent studies on the major cellular responses induced by peroxisomal dysfunction and the role of inter-organelle crosstalk in maintaining cellular homeostasis and organelle functions. Lastly, we discuss the potential roles of peroxisomes in aging and aging-related diseases.

## 2. Peroxisome Biogenesis and Peroxisomal Import Machinery

Peroxisomes are single membrane-bound organelles found in all eukaryotic cells and organisms, from yeast to plants and mammals [15]. Under normal conditions, the peroxisome has a half-life of 1.5 to 2 days [16], suggesting that peroxisome biogenesis and degradation are very dynamic processes. Peroxisomal biogenesis is mostly regulated by peroxins encoded by *PEX* genes. To date, more than 30 conserved peroxins have been identified in yeast, plants, and many animal species [17]. Peroxisomes can be formed by *de novo* biogenesis from the ER and mitochondria [17,18,19]. Pre-peroxisomal vesicles originating from the ER contain the peroxisomal membrane protein (PMP) PEX16, which binds to the PMP receptor PEX3. PEX3 functions as a docking receptor for the cytosolic chaperone and PMP receptor PEX19 [20,21]. Together, these peroxins recruit other PMPs containing membrane peroxisome targeting signals (PTS) to the peroxisomal membrane during the peroxisome maturation process.

Peroxisomal matrix proteins are transported into the organelle after synthesis in the cytosol [22]. PTSs are essential for the correct sorting of matrix proteins [23]. Most peroxisomal matrix proteins contain a PTS type 1 (PTS1) sequence at the C-terminus consisting of an S-K-L motif or a conservative variant [24,25]. A small set of matrix proteins contains a PTS type 2 (PTS2) sequence, a nonapeptide with the consensus sequence (R/K)-(L/I/V)-X_5_-(H/Q)-(L/A) (X = any amino acid) that is localized near or at the *N*-terminus of the protein [26,27]. PTS1 proteins are transported to the peroxisome by the shuttling receptor PEX5, whereas PTS2 proteins are delivered to the organelle by PEX5 and PEX7 [23]. The cargo-loaded receptor proteins bind to the peroxisomal membrane proteins PEX13 and PEX14 [28,29]. After docking, the cargo proteins are translocated across the peroxisomal membrane and imported into the organelle lumen, while receptor protein PEX5 is then mono- or polyubiquitinated by E3 ubiquitin ligases PEX2, PEX10, and PEX12 [23,30,31]. PEX5 monoubiquitination is required for its recycling to the cytosol, while polyubiquitinated PEX5 can be targeted to the proteasome for degradation [32,33,34]. Cytosolic AAA-ATPases PEX1 and PEX6 form a heterohexameric ring as a trimer of PEX1/PEX6 heterodimers, which is anchored to the peroxisome membrane by PEX26 in higher organisms [35,36,37,38,39]. The PEX1-PEX6-PEX26 complex is responsible for releasing ubiquitinated PEX5 from the peroxisomal membrane to the cytosol [33,34,35]. In the cytosol, PEX5 can be deubiquitinated by deubiquitylating (DUB) enzymes (Ubp15 in yeast [40] and USP9X in mammals [41]) for the next cycle of peroxisomal import.

Mature peroxisomes divide and grow by elongation, membrane constriction, and fission [22,42,43,44,45]. In mammals, the peroxisomal membrane protein PEX11β plays a critical role in peroxisomal proliferation [42,46,47]. Exogenous expression of *PEX11*β in mammalian cells enhances peroxisomal proliferation [42], whereas the genetic defects of human *PEX11β* decrease peroxisomal abundance [47]. Although the molecular mechanisms underlying the modulation of PEX11β activity are not fully understood, docosahexaenoic acid (DHA) is known to play an important role in the initiation of peroxisomal elongation and fission [48]. Itoyama et al. [48] demonstrated that DHA, a peroxisomal β-oxidation metabolite, induced peroxisomal division by augmenting the hyper-oligomerization of Pex11pβ and the formation of Pex11pβ-enriched regions on elongated peroxisomes. The function of Pex11β in peroxisome proliferation depends on the homo-oligomerization through its *N*-terminal domain [46,49]. In addition, Pex11β interacts with the membrane via the *N*-terminal amphipathic helix to deform the membrane into elongated tubular peroxisomes [50,51,52]. Subsequently, Pex11β forms a ternary fission machinery complex with mitochondrial fission factor (Mff) and dynamin-like protein 1 (DLP1) at the constricted membrane region of elongated peroxisomes, promoting the scission of the membrane [51,53].

## 3. Key Metabolic Functions of Peroxisome

Peroxisomes are multifunctional organelles that play essential roles in several key metabolic pathways, including the β-oxidation of very-long-chain fatty acids (VLCFAs, >C22), the α-oxidation of branched-chain fatty acids, the oxidation of D-amino acids and polyamines, and the synthesis of ether phospholipids, bile acids, and DHA [2,17]. During these metabolic processes (e.g., VLCFA β-oxidation), reactive oxygen species (ROS) are produced as byproducts and subsequently neutralized by several antioxidant enzymes such as catalase [54,55,56]. About 50 different enzymes are involved in peroxisomal metabolic functions [2,57], and some of them are shared with other subcellular compartments, including mitochondria [57]. Accordingly, cooperation with other organelles, including mitochondria, ER, lipid droplets, and lysosomes, is necessary for proper peroxisomal metabolic functions.

Most fatty acids (FAs) are catabolized by β-oxidation, which removes two carbons from their carboxyl terminus [58]. In all eukaryotic cells, fatty acid oxidation occurs in both mitochondria and peroxisomes. FAs (<C20) are oxidized in mitochondria and converted to CO_2_ and H_2_O through the mitochondrial citric acid cycle and oxidative phosphorylation. However, VLCFAs cannot be oxidized in mitochondria. Instead, VLCFAs translocate across the peroxisomal membrane via ATP-binding cassette (ABC) subfamily D half-transporters (ABCD) and are broken down to acetyl-CoA, propionyl-CoA, or medium-chain acyl-CoAs. These different acyl-CoAs can be further converted into acylcarnitines in the peroxisome and translocate to mitochondria through the mitochondrial carnitine/acylcarnitine translocase (CACT). Acylcarnitines are then converted back to acyl-CoAs in mitochondria and enter the citric acid cycle [59].

Ether phospholipids are particular classes of phospholipids containing a glycerol backbone with an ether or vinyl-ether bond at the *sn-1* position [60]. Plasmalogen is one type of ether phospholipid containing a vinyl-ether bond that is widely found in the brain, heart, and white blood cells [17]. The biosynthesis of plasmalogen begins in peroxisomes [17,60,61]. Dihydroxyacetone (DHAP), a glycolysis intermediate, is used as a building block of the glycerol backbone of plasmalogen. The peroxisomal matrix enzyme glyceronephosphate *O*-acyltransferase (GNPAT) transfers the acyl chain of acyl-CoA to DHAP to generate acyl-DHAP. Then, alkyl dihydroxyacetone phosphate synthase (AGPS) exchanges the acyl chain for an alkyl group to form alkyl-DHAP. Recently, Honsho et al. [62] showed that acyl/alkyl-DHAP reductase (ADHAPR/DHRS7B) localizes in peroxisomes and the ER as a type I integral membrane protein in HeLa cells. The authors demonstrated that ER-localized ADHAPR reduces alkyl-DHAP, while peroxisomal ADHAPR preferentially reduces acyl-DHAP before the subsequent synthesis of alkyl-DHAP catalyzed by alkyl-DHAP synthase (ADAPS) [62]. The remaining steps of plasmalogen synthesis take place in the ER [63,64].

Peroxisomes are also important for ROS metabolism. The name “peroxisome” was first introduced by De Duve in 1965 to define an organelle containing several enzymes that produce or break down hydrogen peroxide (H_2_O_2_) [4,65]. Peroxisomes are responsible for up to 20% of the cell’s oxygen consumption and 35% of the H_2_O_2_ production in mammalian tissues [66]. Peroxisomes produce different ROS species such as superoxide, hydroxyl radicals, and hydrogen peroxide as byproducts of metabolic processes by multiple oxidases present in the organelle. Acyl-CoA oxidases (ACOX) are the most abundant ROS-generating enzymes in the peroxisome [59]. D-amino acid oxidase (DAO), D-aspartate oxidase (DDO), L-pipecolic acid oxidase (PIPOX), L-α-hydroxy acid oxidases (HAO), polyamine oxidase (PAOX), and xanthine oxidase (XDH) are also important H_2_O_2_ producers in human peroxisomes. To protect the organelle from oxidative damage, human peroxisomes have several antioxidant enzymes including catalase (CAT), copper/zinc-containing superoxide dismutase (SOD1), peroxiredoxin 5 (PRDX5), glutathione S-transferase kappa 1 (GSTK1), microsomal GST1 (MGST1), and epoxide hydrolase 2 (EPHX2) [67].

## 4. Cellular Responses to Peroxisomal Dysfunction

**Figure 1:**
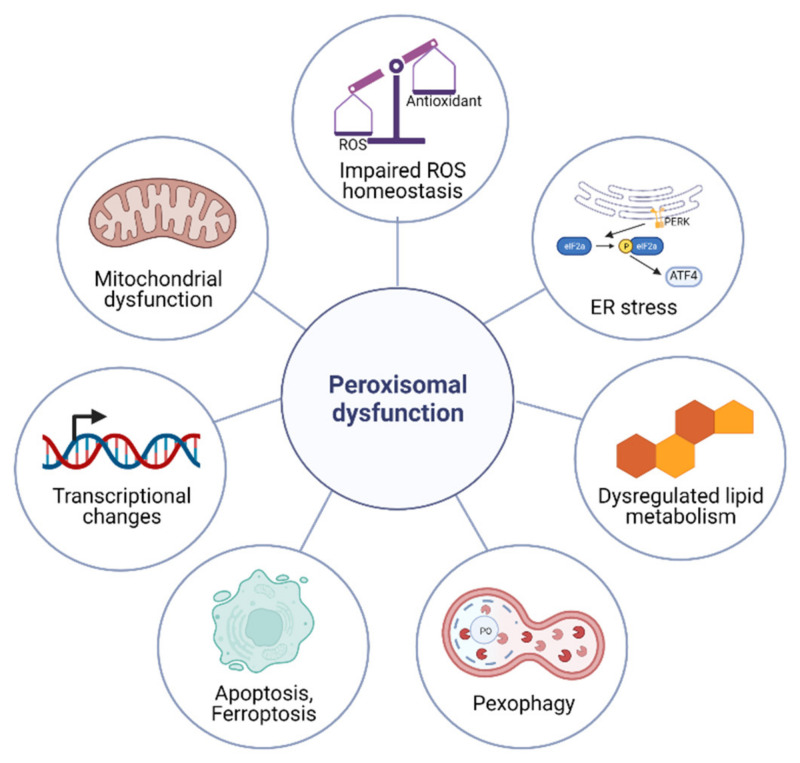
Cellular responses to peroxisomal dysfunction. Impaired peroxisomal function elicits cellular responses such as the impaired ROS homeostasis, dysregulated lipid metabolism, mitochondrial dysfunction, altered gene expression, pexophagy, elevated ER stress, and apoptosis.

The metabolic functions of peroxisomes are critical for cellular homeostasis, and the impairment of proper peroxisomal function elicits a variety of cellular responses (Figure 1). Here, we propose that there is a signaling pathway that is responsible for sensing defective peroxisomes and activating cytoprotective mechanisms, which we coin as the peroxisomal stress response pathway. In this section, we categorize peroxisome stress responses into seven categories: transcriptional changes, impaired ROS homeostasis, dysregulated lipid metabolism, mitochondrial dysfunction, ER stress, apoptosis and ferroptosis, and pexophagy (Figure 1).

### 4.1. Transcriptional Changes upon Peroxisomal Dysfunction

Several lines of evidence suggest that an uncharacterized cellular stress response pathway might exist in eukaryotic cells to cope with peroxisomal dysfunction. Recent studies have revealed global transcriptional changes in PEX mutants with impaired peroxisomal import [9,68,69,70,71,72]. Peeters et al. [68] investigated the role of peroxisomes in liver carbohydrate metabolism through a microarray analysis using hepatocyte-specific *Pex5* knockout mice (*L-Pex5^−/−^* mice). The depletion of *Pex5* in hepatocytes significantly suppressed the mRNA levels of two enzymes involved in gluconeogenesis (*Pck1*, *G6pc*) as well as the mRNA level of glycogen synthase 2 (*Gys2*). However, some glycolysis genes (e.g., *Aldoa*, *Pdk4*) were upregulated in the *Pex5* knockout hepatocytes. Follow-up experiments demonstrated that peroxisomal and mitochondrial abnormalities trigger energy deficits, evidenced by increased cellular AMP/ATP ratios and decreased NAD^+^/NADH ratios. This causes AMP-activated kinase (AMPK) activation and peroxisome proliferator-activated receptor γ coactivator 1α (PGC-1α) suppression, resulting in the down-regulation of glycogen synthesis and the induction of glycolysis [68].

A recent transcriptome analysis was carried out to study the role of peroxisomes in pancreatic β-cell homeostasis using β-cell-selective *Pex5* knockout mice (*Rip-Pex5*^−/−^) [69]. Similar to *Pex5* knockout hepatocytes (L-*Pex5*^−/−^) [73], *Pex5*-deficient pancreatic β-cells exhibited abnormal mitochondrial shape and reduced complex I activity [69]. The genes involved in apoptosis and β-cell dysfunction, including *Cdkn1a*, *Pmaip11*, *Pidd1*, *Txnip*, *Pitgs*, and *Thbs2*, were up-regulated in the pancreatic β-cells of *Pex5* knockout mice. The expression levels of genes with potential anti-apoptotic effects gene, including *Sfrp1* and the insulin secretion promoting gene *Alox5*, were enhanced in islets of *Rip-Pex5^−/−^* mice, possibly as a compensatory mechanism [69].

X-linked adrenoleukodystrophy (X-ALD) is a peroxisomal disorder characterized by axonopathy and demyelination in the central nervous system and adrenal insufficiency [74]. The disease is caused by mutations in the *ABCD1* gene mapped to chromosome Xq28, which functions as a transporter of VLCFAs and VLCFA-CoA esters into the peroxisome for β-oxidation [70,74]. The classical mouse model of X-ALD is the *Abcd1* gene knockout (*Abcd*1^−/−^) [75]. Schluter et al. [71] performed a microarray-based functional genomics analysis of the spinal cord of *Abcd1* knockout mice which revealed that several key metabolic and cell signaling pathways are involved in X-ALD pathogenesis. Based on this transcriptome analysis, the metabolic and cell signaling pathways involved in mitochondrial dysfunction, ROS production with antioxidant defense impairment, insulin and adipocytokine signaling dysregulation, impaired protein synthesis and turnover, as well as NF-κB mediated pro-inflammatory responses were found to play a potential role in X-ALD pathogenesis [71].

Catalase is an essential antioxidant enzyme predominantly located within peroxisomes that catalyzes the conversion of hydrogen peroxide to oxygen and water. The global knockout of catalase in mice (*Cat*^−/−^) resulted in increased oxidative stress and body weight [72]. The transcriptome analysis of liver tissue from *Cat*^−/−^ mice revealed the differential expression of many metabolic genes, especially those involved in lipid metabolism. For example, catalase knockout resulted in the up-regulation of adipsin (*Cfd*), cell death-inducing DFFA-like effector C (*Cidec*), oxysterol binding protein-like 3 (*Osbpl3*), protein phosphatase 1 regulatory subunit 3G (*Ppp1r3g*), monoacylglycerol acyltransferase (*Mogat1*), fatty acid translocase (*Cd36*), fatty acid synthase (*Fasn*), endothelial lipase (*Lipg*), stearoyl-CoA desaturases (*Scd1*, *Scd2*, *Scd3*, *Scd4*), and alpha1A adrenoceptors (*Adra1a*). At the same time, the knockout of catalase down-regulated lipoprotein lipase (*Lpl*), beta2 adrenoceptors (*Adrb2*), and 3-hydroxy-3-methylglutaryl-CoA reductase (*Hmgcr*). In addition, the loss of catalase influenced the actions of insulin, down-regulating genes encoding insulin-like growth factor binding protein 1 (*Igfbp1*) and insulin receptor substrate 2 (*Irs2*) and up-regulating the insulin receptor substrate 3 (*Irs3*). These data provide evidence that catalase deficiency plays a vital role in insulin resistance and the development of a pre-diabetic state [72].

Recently, we performed a comparative transcriptomic analysis to interrogate the conserved peroxisomal stress responses between the fruit fly and humans. We generated stable human HEK293 cells with doxycycline-inducible expression of a mutated *PEX5* (PEX5^C11A^), which blocks PEX5 recycling and PEX5-mediated peroxisomal import [9]. PEX5^C11A^ expression decreased the mRNA level of genes involved in mitochondrial oxidative phosphorylation and increased the mRNA level of genes involved in MAPK signaling and Hippo signaling, presumably due to increased ROS production. To identify conserved responses, we performed a transcriptomic analysis on *Drosophila* with oenocyte-specific *Pex1*, *Pex12*, and *Pex5* knockdowns (oenocytes are the fly hepatocytes). Our transcriptome analysis showed that oxidative phosphorylation genes were repressed, and the inflammation pathway was induced upon peroxisomal stress in both fly and human cells. Furthermore, we found that peroxisomal dysfunction in both fly and human cells repressed ribosome biogenesis and activated ER stress response pathways, especially the eIF2α-ATF4 pathway, suggesting potential crosstalk between peroxisomes and the ER [9].

Although further investigations are needed to determine the direct mechanisms underlying peroxisome-nucleus communication, recent transcriptomic studies strongly indicate that peroxisomal dysfunction can directly modulate transcriptional activities in the nucleus.

### 4.2. Impaired ROS Homeostasis

Peroxisomes play critical roles in both the production and scavenging of hydrogen peroxide. Given the essential role of peroxisomes in redox homeostasis, impaired peroxisomal function disrupts cellular ROS homeostasis. Several studies have reported excessive cellular ROS production upon peroxisomal dysfunction. For example, impaired peroxisomal import by knocking down *Pex5* in adipocytes significantly elevated cytosolic ROS production [76]. Likewise, peroxisome disfunction associated with *Pex13* [77] or *Pex11b* [78] deficiency led to higher ROS production in lung fibroblasts and neurons, respectively.

The major sources of elevated ROS levels upon peroxisomal dysfunction remain elusive. Presumably, mitochondria are the major contributor to overall cellular oxidative stress under peroxisomal impairment. Multiple studies have shown that mitochondrial ROS are elevated by peroxisomal dysfunction. For example, Walton and Pizzitelli [79] demonstrated that altered peroxisomal oxidative balance associated with the inhibition of catalase activity increased mitochondrial ROS production and decreased the activity of the mitochondrial enzyme aconitase, an enzyme that participates in the Krebs Cycle and is sensitive to oxidative stress. Moreover, the inhibition of catalase activity decreased the inner membrane potential in mitochondria, suggesting that ROS induced by impaired peroxisomal function contribute to mitochondrial dysfunction [79]. Importantly, Ivashchenko et al. [80] demonstrated that peroxisome-derived oxidative stress disturbed the mitochondrial redox balance. In their study, Ivashchenko et al. artificially induced the generation of excessive ROS exclusively inside peroxisomes by employing Killer Red, a genetically encoded photosensitizer that produces radicals and H_2_O_2_ upon green light illumination. In combination with a redox-sensitive green fluorescent protein (roGFP), the authors found that mitochondrial redox balance was disturbed by peroxisome-derived oxidative stress [80].

Whether animals with catalase deficiency show elevated ROS production inside peroxisomes themselves remains unclear. Hwang et al. [81] showed that the inhibition of catalase in hepatocytes significantly increased the peroxisomal H_2_O_2_ level, as measured by a peroxisome-targeted hydrogen peroxide probe (Hyper-P). However, another study reported that the redox environment of the peroxisomal matrix was not increased in catalase-deficient mouse embryonic fibroblast (MEF) cells [80]. The study measured the oxidation state using peroxisome-specific (roGFP2-PTS1) and mitochondria-specific (mt-roGFP2) ROS-detecting fusion proteins and found that only the redox status of mitochondria was significantly increased by either knocking out catalase activity or treating cells with the catalase inhibitor 3-amino-1,2,4-triazole (3-AT).

### 4.3. Dysregulated Lipid Metabolism

Peroxisomes contribute to cellular lipid metabolism including the β-oxidation of VLCFAs and the biosynthesis of cholesterol, bile acids, PUFAs, and ether phospholipids. Given the crucial roles of peroxisomes in lipid metabolism, accumulating evidence shows that lipid metabolism is largely altered in response to the impairment of peroxisomes.

VLCFAs are exclusively catabolized into a range of chain-shortened acyl-CoAs by peroxisomal β-oxidation, and the resulting acetyl-CoA, propionyl-CoA, and different medium-chain acyl-CoAs are delivered to mitochondria for further oxidation to CO_2_ and H_2_O [59]. Therefore, elevated plasma VLCFAs are associated with the clinical diagnosis of peroxisomal disorders, including X-linked adrenoleukodystrophy (X-ALD) [82,83]. Moser et al. [82] reported elevated plasma levels of VLCFA in patients with X-ALD, ZS, neonatal adrenoleukodystrophy (NALD), infantile Refsum’s disease (IRD), and in patients with deficiencies of peroxisomal acyl-coenzyme A oxidase (ACOX1), D-bifunctional protein (DBP), and 3-oxoacyl-coenzyme A thiolase. The levels of VLCFA in these patients were correlated with the severity of the disease [82]. Another study [84] showed that high levels of serum C26:0 were associated with a short survival time in patients with ZS, DBP, and mild ZS. However, it should be noted that dietary restriction of VLCFA in combination with oral supplementation with Lorenzo’s oil (a 4:1 mixture of glycerol trioleate and glycerol trierucate) reduced VLCFA plasma levels but did not result in clinical improvement [85] nor did it arrest the progression of the disease in X-ALD patients [86], suggesting that the correlation between serum VLCFA levels and clinical severity may not be direct.

Peroxisome deficiency mouse models recapitulate dysregulated lipid metabolism phenotypes seen in human PBD patients [87]. *Pex2* knockout mice showed 10-fold increased plasma levels of VLCFAs [88]. *Pex13* [89] and *Pex5* [90] knockout mice exhibited increased levels of VLCFA in liver and brain tissue compared to wild-type animals. Additionally, the levels of plasmalogen, PUFAs, and DHA were significantly decreased in newborn *Pex5* knockout mice compared to normal littermates [91]. *Pex7* knockout mice also showed plasmalogen deficiency. Alkyl-glycerols supplementation to *Pex7^−/−^* mice restored plasmalogen levels in erythrocytes and several tissues including the kidney, heart, and eye, whereas only a marginal increase was observed in nervous tissues [92]. Hofer et al. [93] showed that peroxisomal fatty acid oxidation was decreased by the knockdown of Pex16 in adipocytes, resulting in the accumulation of VLCFAs and reduced levels of peroxisomal α-oxidation derived odd-chain fatty acids. Furthermore, the cellular oxygen consumption rate was reduced upon Pex16-silencing, suggesting impaired mitochondrial β-oxidation. These findings support the critical role of peroxisomes in lipid homeostasis.

Other model organisms show similar lipid metabolism phenotypes upon the loss of peroxisomes. Rackles et al. [10] showed that the knockdown of *prx-5/PEX5* in *Caenorhabditis elegans* (*C. elegans*) resulted in increased levels of triacylglycerols with long acyl chains, suggesting reduced β-oxidation activity. In addition, the depletion of *prx-5* resulted in reduced biosynthesis of ether phospholipids, which are generated exclusively in peroxisomes. Sellin et al. [94] reported that peroxisome loss in a *Drosophila Pex19* mutant (*Pex19^ΔF7^*) was associated with the accumulation of VLCFAs.

Cholesterol is a major structural component of cellular membranes, and it serves as a precursor molecule for the synthesis of steroid hormones and bile acids [95]. The biosynthesis of cholesterol consists of multiple processes, beginning with acetyl-CoA. Plasmalogen and acetoacetyl-CoA are converted to 3-hydroxy-3-methylglutaryl-CoA (HMG-CoA) by HMG-CoA synthase. HMG-CoA is then converted to mevalonate by HMG-CoA reductase. Mevalonate is metabolized to farnesyl-diphosphate (farnesyl-PP) by a series of enzymatic reactions catalyzed by enzymes such as mevalonate kinase, phosphomevalonate kinase, mevalonate-PP decarboxylase, isopentenyl-PP isomerase, and farnesyl diphosphate synthase (for the comprehensive steps of cholesterol biosynthesis, see review [96]). All of the enzymes that convert acetyl-CoA to farnesyl-PP are cytosolic enzymes, except for HMG-CoA reductase, which is localized in the ER.

Several lines of evidence suggest that peroxisomes not only have a role in cholesterol oxidation but also in cholesterol biosynthesis. Krisans et al. [97] found that the liver tissue of ZS patients showed significantly decreased activities of enzymes involved in cholesterol biosynthesis, including HMG-CoA reductase, mevalonate kinase (MVK), and phosphomevalonate kinase. In line with these observations, Kovacs et al. [95] showed that *Pex2* knockout mice (*Pex2*^−/−^) had decreased plasma levels of total and high-density lipoprotein cholesterol compared to wild-type mice. The cholesterol content in the liver tissue of *Pex2*^−/−^ mice was reduced by about 40% relative to control mice. A follow-up study revealed that the loss of peroxisomes activates ER stress, which leads to the dysregulation of the endogenous sterol response pathway [98].

However, the role of peroxisomes in cholesterol biosynthesis is still debatable. Hogenboom et al. [99] found that the protein levels and activities of key enzymes of cholesterol biosynthesis in the liver homogenates of *Pex5* knockout mice were normal. Likewise, cholesterol biosynthesis was not defective in the fibroblasts of ZS patients [100]. In addition, in contrast to the results obtained by Krisans et al. [97], Hogenboom et al. [101,102] found that mevalonate kinase, phosphomevalonate kinase, and mevalonate pyrophosphate decarboxylase were cytosolic, not peroxisomal enzymes.

Peroxisomal dysfunction also affects intracellular cholesterol trafficking from lysosomes to peroxisomes. Chu et al. [103] identified a number of proteins related to peroxisomal function and biogenesis that are required for the transport of low-density lipoprotein-derived cholesterol through genome-wide pooled shRNA screening. The disruption of critical peroxisomal genes, such as *ABCD1* and *PEX1*, resulted in significant decreases in plasma membrane cholesterol levels and the accumulation of cholesterol in lysosomes. These findings reveal an unexpected role of peroxisomes in intracellular cholesterol transport. Furthermore, massive cholesterol accumulation was observed in human patient cells and mouse models of peroxisomal disorder [7,103], demonstrating the clinical relevance of cholesterol transport in peroxisomal disorders.

### 4.4. Mitochondrial Dysfunction

It has become clear that peroxisomes and mitochondria have a strong interconnection, as they show complementary activities and exchange many metabolites [59,73,104]. The coordinated interplay between peroxisomes and mitochondria is required for diverse metabolic and cellular processes such as the β-oxidation of fatty acids, redox homeostasis, as well as inflammatory and innate immune responses [104]. In line with the close relationship between the two organelles, multiple studies have reported that the loss of functional peroxisomes causes mitochondrial dysfunction [73,89,105,106,107,108,109,110].

Mitochondrial abnormalities have been widely reported in ZS patients. Although the detailed mechanisms remain unsolved, ZS patients exhibit altered structure of the inner mitochondrial membrane and decreased activities of respiratory chain complexes [111,112,113,114]. Accordingly, the depletion of functional peroxisomes in a *Pex5* knockout mouse model (L-*Pex5*^−/−^) exhibiting selective elimination of liver peroxisomes resulted in mitochondrial abnormalities characterized by an altered inner mitochondrial membrane with twisted or irregular cristae, a dense matrix, and crystalline inclusions [106]. The ultrastructural changes of the mitochondrial inner membrane in *Pex5* knockout mice were similar to those observed in ZS patients [114]. In addition, the knockout of *Pex2* [107] or *Pex13* [89] resulted in swollen mitochondria with abnormally shaped cristae in hepatocytes [73]. Besides the morphological changes, the depletion of *Pex5* in hepatocytes reduced mitochondrial DNA content, inhibited the activities of the respiratory chain complex I, III, and V, reduced membrane potential, and increased ROS production [73].

Peroxisomal dysfunction has recently been linked to altered mitochondrial dynamics. Park et al. [110] studied the role of peroxisomes in mitochondrial dynamics and thermogenesis in adipose tissue. The authors demonstrated that the adipose-specific knockout of *Pex16* in mice disrupted cold-induced mitochondrial fission, decreased the expression of mitochondrially encoded genes involved in oxidative phosphorylation, and reduced the rate of coupled and uncoupled respiration [110]. In *Drosophila*, *Pex19* mutants showed increased mitochondrial size compared to wild-type flies and had increased levels of etomoxir-sensitive oxygen consumption, suggesting enhanced mitochondrial β-oxidation rates [109].

### 4.5. Endoplasmic Reticulum (ER) Stress

The ER is the site where secretory and membrane proteins are synthesized, folded, and matured. The accumulation of unfolded or misfolded proteins can activate the ER stress response. In mammalian cells, there are three ER stress pathways mediated through three stress sensors: protein kinase RNA-like ER kinase (PERK), inositol-requiring protein 1 (IRE1), and activating transcription factor 6 (ATF6) [115]. Upon ER stress, PERK phosphorylates eIF2α, leading to translation inhibition and the activation of the transcription factor ATF4 [116,117,118]. On the other hand, membrane-bound RNase IRE1 splices the transcription factor XBP1 pre-mRNA into its mature form, which is translocated to the nucleus to induce the transcription of ER-associated degradation (ERAD) genes [119]. Lastly, the transcription factor ATF6 is transported to the Golgi apparatus and sequentially cleaved by proteases such as S1P and S2P. The mature ATF6 translocates to the nucleus to induce the expression of ER chaperone genes [120].

Several recent studies reported that the impairment of peroxisomes induces ER stress [98,121,122,123]. Kovacs et al. [98] provided the first evidence that *Pex2* knockout activated hepatic ER stress pathways in mice by showing increased mRNA expression of *ATF4* and its transcriptional targets, such as *ATF3, CHOP,* and *TRIB3*. Another study from the same group showed that the phosphorylation of eIF2α was highly induced in newborn *Pex2* knockout mice [121]. Accordingly, the silencing of *PEX2* in human liver hepatocarcinoma cell lines induced the mRNA expression of ER stress markers such as *PERK, Erol-Lα, PDI,* and *CHOP* [122]. Our recent study also supports the link between peroxisomal dysfunction and ER stress [9]. We found that the ER-associated protein degradation (ERAD) pathway was induced in *Pex1*, *Pex5*, and *Pex12* knockdowns in *Drosophila.* Similar to mouse studies, the phosphorylated eIF2α was significantly elevated in both peroxin knockdown flies and human *PEX5^C11A^* mutant cells. Interestingly, we observed that the level of spliced XBP1 was not altered in both *Pex5* knockdown flies and *PEX5^C11A^* mutant human cells. Similarly, IRE1α signaling and its RNase activity were not significantly induced in the liver of *Pex2* knockout mice [98]. Thus, these results suggest that peroxisomal deficiency activates a specific branch of ER stress pathways. It seems that the PERK-eIF2α-ATF4 branch, but not IRE1α-XBP1, is targeted by peroxisomal dysfunction. As eIF2α-ATF4 signaling is also involved in the integrated stress response (ISR) pathway, peroxisomal dysfunction can likely activate the ISR pathway. It has been proposed that ER stress might be induced by the metabolic changes associated with hepatic *Pex2* knockout, such as the perturbed flux of mevalonate metabolites, dysregulated bile acid homeostasis, altered fatty acid levels and composition, and oxidative stress [123]. However, the exact mechanisms by which peroxisomal dysfunction controls ER stress and ISR signaling pathways remain to be determined.

### 4.6. Apoptosis and Ferroptosis

Peroxisomal dysfunction has been linked to apoptotic cell death. Peroxisome-deficient mice with *Pex5* mutations showed elevated apoptotic cell death in the white matter and the cortical plate [90]. Transcriptomic profiling of *Pex5* knockout pancreatic β-cells revealed the up-regulation of genes involved in apoptosis such as *Cdkn1a*, *Pmaip11*, and *Pidd1*. Results from the TUNEL assay further confirmed that apoptosis was increased in the β-cells of *Pex5* mutants [69]. Similarly, knockout of *Pex5* in the developing mouse cerebellum showed significantly higher numbers of caspase-3-positive cells, compared to control littermates [124]. Peroxisomal dysfunction associated with *Pex16* deficiency in human skin fibroblast cells resulted in increased apoptotic cell death [125]. In addition, mouse embryonic fibroblast (MEF) with either *Pex3* or *Pex5* knockout showed elevated cytochrome c levels in the cytoplasm and an increase in the amounts of cleaved caspase-9 and caspase-3. The cell death phenotypes were rescued by the overexpression of *Pex3* or *Pex5* in the corresponding knockout MEFs, suggesting that peroxisomal dysfunction modulates caspase activity and apoptosis [126].

Recently, peroxisomes were shown to play an important role in ferroptosis, a unique process of nonapoptotic cell death caused by the accumulation of iron-dependent lipid peroxides [127]. Ferroptosis differs from other forms of cell death including apoptosis because it does not require caspase activation. Instead, increased activity of lipoxygenase and cytochrome P450 oxidoreductase is responsible for lipid peroxidation. In particular, the oxidation of PUFAs is a key driver of ferroptosis [127]. In a recent genome-wide CRISPR screen combined with lipidomic profiling, Zou et al. [128] reported that polyunsaturated ether phospholipids (PUFA-ePLs) synthesized in the peroxisome act as substrates for lipid peroxidation, resulting in the induction of ferroptosis in human renal and ovarian carcinoma cells. Carcinoma cells can evade ferroptosis through the downregulation of PUFA-ePLs. Although plasmalogens were previously described as cellular antioxidants, specific PUFA-ePLs may be pro-ferroptotic and act as key regulators in ferroptosis-associated pathologies [128].

### 4.7. Autophagy and Pexophagy

Peroxisomal homeostasis is maintained by the coordination of peroxisomal biogenesis and degradation. Peroxisomes have a half-life of 1.5 to 2 days, suggesting that peroxisomal homeostasis is very dynamic [129]. In mammals, three independent mechanisms have been proposed for peroxisome degradation, including Lon protease-mediated matrix protein removal, 15-lipoxygenase-mediated autolysis, and pexophagy [130]. Peroxisomal Lon protease 2 (LONP2) is a homo-oligomeric ATP-dependent protease with chaperone-like activity, and LONP2 is responsible for the degradation of excessive and unnecessary matrix proteins, such as β-oxidation enzymes [131,132]. 15-lipoxygenase (15-LOX) is responsible for the autolysis of the peroxisomal membrane by the peroxidation of membrane lipids [133].

However, the major degradation pathway of peroxisomes is pexophagy. Pexophagy is a type of macroautophagy, and 80% of peroxisomes are removed by this process [134]. Like other macroautophagy processes, pexophagy includes three major steps: (1) autophagy receptors NBR1 and p62 recognize ubiquitinated peroxisomal membrane proteins, (2) target peroxisomes are sequestered by autophagosome, and (3) sequestered peroxisomes are delivered to the lysosome for degradation [135].

The ubiquitination of PEX5 plays a key role in maintaining peroxisome homeostasis. Under normal conditions, PEX5 is usually ubiquitinated at conserved cysteine 11 residue by the RING (Really Interesting New Gene) E3 ligase complex that consists of PEX2, PEX10, and PEX12. Then, monoubiquitinated PEX5 facilitates its release from peroxisomes by the AAA-ATPase complex (PEX1, PEX6, and PEX26). The loss of peroxisomal AAA-complex function is commonly found in peroxisomal biogenesis disorders (PBDs) [136]. PBD patients with mutations in the AAA-complex show decreased peroxisomal number and function [2]. Law et al. [137] demonstrated that the loss of AAA-complex function in mammalian cells resulted in the accumulation of ubiquitinated PEX5 on the peroxisomal membrane that triggers peroxisomal degradation by NBR1-dependent pexophagy. In addition, the authors showed that autophagy inhibitors restored peroxisome number and matrix protein import in *PEX1*-mutated PBD fibroblasts, suggesting that the peroxisomal AAA-complex is required for peroxisomal quality control and the regulation of pexophagy [137]. However, conflicting findings were recently reported. Klouwer et al. [138] found that autophagy inhibitors, including hydroxychloroquine, did not improve peroxisomal metabolic functions and peroxisomal matrix protein import but, in fact, caused a further decrease of peroxisomal functions in the primary skin fibroblast cells of PBD patients. Thus, further investigations are needed to confirm the beneficial roles of autophagy inhibitors in PBD treatment [138].

Interestingly, the loss of peroxisomal function not only induces pexophagy but also alters other macroautophagy processes, such as lipophagy [139]. In the lipid metabolic process, besides cytosolic lipolysis, triglycerides can be hydrolyzed by lipophagy. Lipophagy plays a crucial role in lipid degradation through the engulfment of lipid droplets within the autophagosomes and hydrolysis of triglycerides by lysosomal acid lipase A (LIPA/LAL) to free fatty acid and glycerol [140,141]. Recently, He et al. [139] demonstrated that hepatocyte-specific knockout of the peroxisomal β-oxidation enzyme *Acox1* induced autophagic degradation of lipid droplets and protected mice from hepatic steatosis induced by starvation or a high-fat diet. These results suggest that peroxisome-derived acetyl-CoA plays an important role in the regulation of lipophagy.

The role of peroxisomes in the regulation of the autophagic degradation of other organelles has just started to be uncovered. To date, several selective autophagic pathways have been discovered: mitophagy (mitochondria), proteaphagy (proteasomes), ribophagy (ribosomes), ER-phagy (ER), lysophagy (lysosome), nucleophagy (nuclei), and pexophagy (peroxisome) [142]. Since peroxisomes are functionally connected with other organelles, it would be interesting to further investigate whether and how peroxisomal dysfunction affects selective autophagic processes in organelle clearance and homeostasis.

## 5. Peroxisome-Organelle Communication

**Figure 2:**
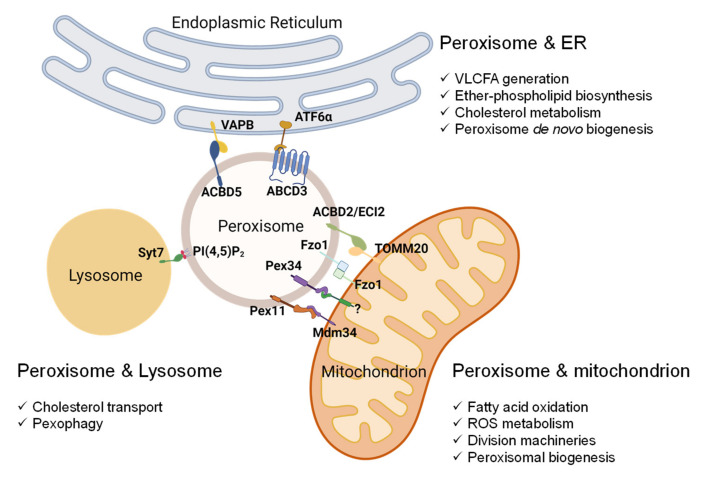
Peroxisome-organelle membrane contact sites and their roles in organelle functions. Peroxisomes’ contact with mitochondria involves (1) peroxisomal Pex11 and mitochondrial Mdm34, (2) peroxisomal Pex34 and an unknown mitochondrial protein, (3) mitofusin Fzo1 on both peroxisome and mitochondria, and (4) peroxisomal ACBD2/ECI2 and mitochondrial TOMM20. The interaction between peroxisomes and the ER involves (1) peroxisomal ACBD5 and resident ER protein VAPB and (2) peroxisomal ABCD3 and ER protein ATF6α upon Ceapin treatment. In mammalian cells, the contact between peroxisomes and lysosomes involves phospholipid PI(4,5)P_2_ on the peroxisomal membrane and lysosomal Syt7. Abbreviations: Pex11, peroxin 11; Mdm34, mitochondrial distribution and morphology protein 34; Pex34, peroxin 34; Fzo1, mitofusin; ACBD2/ECI2, acyl-coenzyme A-binding domain or enoyl-CoA-δ isomerase 2; TOMM20, translocase of outer mitochondrial membrane 20; ACBD5, acyl-CoA binding domain-containing protein 5; VAPB, vesicle-associated membrane protein-associated protein B/C; ABCD3, ATP binding cassette subfamily D member 3; ATF6, activating transcription factor 6; PI(4,5)P_2_, phosphatidylinositol 4,5-bisphosphate; Syt7, synaptotagmin VII.

**Figure 3:**
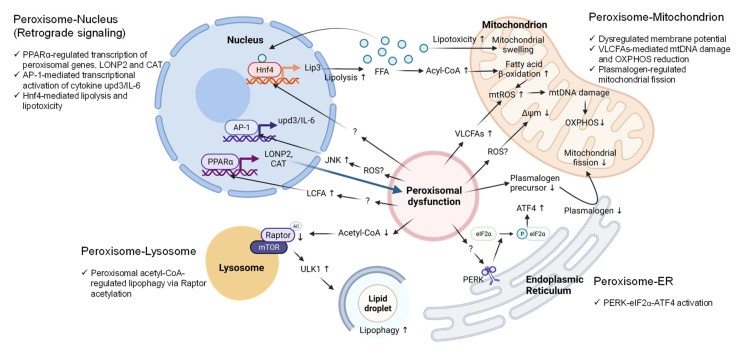
Metabolic and molecular signaling pathways mediate peroxisome-organelle crosstalk in response to peroxisomal dysfunction. (1) Peroxisome-nucleus: Peroxisomal dysfunction activates nuclear gene transcription through the transcription factors PPARα, AP-1, or Hnf4. The accumulation of long-chain fatty acids (LCFAs) activates PPARα to upregulate LONP2 and CAT expression to restore peroxisomal function (Blue arrow). Elevated ROS activates JNK signaling and AP-1 to induce the transcription of the pro-inflammatory cytokine *IL-6*. Peroxisomal dysfunction activates Hnf4 through unknown mechanisms to induce the expression of *lipase 3 (Lip3)*, which leads to excessive free fatty acids (FFA) in the cytoplasm. FFAs hyper-activate Hnf4 via a positive feedback loop. (2) Peroxisome-mitochondrion: Peroxisomal dysfunction causes mitochondrial swelling, mitochondrial ROS generation, mitochondrial DNA (mtDNA) damage, disruption of oxidative phosphorylation (OXPHOS), and decreased inner membrane potential. Excessive ROS and elevated VLCFA are the potential signaling molecules that mediate the peroxisome-mitochondrion crosstalk. Peroxisomal dysfunction decreases plasmalogen, resulting in the inhibition of mitochondrial fission. (3) Peroxisome-ER: Peroxisomal dysfunction activates PERK signaling through unknown mechanisms to increase the phosphorylation of eIF2α and ATF4 induction. (4) Peroxisome-lysosome: Peroxisomal dysfunction due to the loss of Acox1 decreases peroxisome-derived acetyl-CoA, resulting in Raptor acetylation and lysosome localization of mTOR, leading to ULK1 activation and elevated lipophagy. Abbreviations: PPARα, peroxisome proliferator activated receptor alpha; AP-1, activator protein-1; Hnf4, hepatocyte nuclear factor 4; LONP2, lon peptidase 2; CAT, catalase; upd3, unpaired 3; IL-6, interleukin 6; Lip3, lipase 3; ROS, reactive oxygen species; JNK, Jun N-terminal kinase; VLCFAs, very-long-chain fatty acids; PERK, protein kinase RNA-like endoplasmic reticulum kinase; eIF2α, eukaryotic translation initiation factor 2a, ATF4, activating transcription factor 4; mTOR, mechanistic target of rapamycin kinase; ULK1, Unc-51-like autophagy-activating kinase 1.

Peroxisomes are multifunctional and dynamic organelles that actively contribute to cellular signaling, cell fate, immunity and inflammation, and aging [6]. To accomplish these cellular activities, peroxisomes interact and coordinate with other subcellular organelles, such as mitochondria, ER, lysosomes, and lipid droplets [6,59,104]. The cellular changes that accompany peroxisomal dysfunction, discussed in Section 4, are strongly associated with peroxisome-organelle crosstalk. In this section, we summarize the current knowledge on peroxisome-organelle communication involving membrane contact sites, peroxisome-derived signaling metabolites, and retrograde signaling (Figure 2 and Figure 3). In addition, we highlight some of the potential mechanisms by which inter-organelle communication contributes to peroxisomal stress responses.

### 5.1. Peroxisome-Nucleus Crosstalk

All peroxisomal genes are encoded in the nucleus, and peroxisomal proteins are imported to either the peroxisomal membrane or matrix after well-coordinated transcription and translation processes [143]. In mammals, peroxisomal proliferation and lipid metabolism are regulated by nuclear transcription factors including the nuclear receptor peroxisomal proliferator-activated receptor alpha (PPARα) [144]. PPARα acts as a receptor for various compounds. It can be activated by natural fatty acids, such as acyl-CoAs, oxidized fatty acids, eicosanoids, and phytanic acid. PPARα can also be activated by synthetic compounds such as fibrates [144]. The activation of PPARα induces the expression of genes involved in peroxisomal fatty acid β-oxidation, as well as genes involved in peroxisomal biogenesis (*PEX* genes) [145].

Several transcriptomic studies have revealed that peroxisomal dysfunction triggers global transcriptional changes [9,68,69,70,71,72]. However, how cells relay the peroxisomal stress signal to the nucleus (retrograde signaling) remains largely unclear. Organelle retrograde signaling is well established in the case of mitochondria-nucleus and ER-nucleus crosstalk. A recent study suggests that peroxisomal retrograde signaling might also exist in eukaryotic cells [10]. Rackles et al. [10] found that peroxisomal import stress associated with *prx-5/PEX5* knockdown in *C. elegans* induced the expression of peroxisomal Lon protease *lonp-2/LONP2* and catalase *ctl-2/CAT* through the activation of NHR-49/PPARα and its co-factor MDT-15/MED15. Lipidomic analysis revealed that peroxisomal stress increased the levels of long-chain fatty acids due to reduced peroxisomal β-oxidation. Given that PPARα is activated by natural fatty acids, the authors proposed that excessive long-chain fatty acids associated with peroxisomal import stress might serve as the signal that relays peroxisomal stress to the nucleus [10].

In our recent study, we reported that the hepatocyte-specific knockdown of *Pex5* in *Drosophila* induced the expression of the inflammatory cytokine *upd3*, the fly homolog of mammalian interleukin 6 (IL-6), in a JNK-dependent manner [14]. We further showed that the AP-1 transcription factors induced by *Pex5* knockdown were required for the transcriptional activation of *upd3/IL-6* and systemic inflammation in flies [14]. Elevated ROS in *Pex5* knockdown flies likely activates JNK signaling, which promotes nuclear AP-1 activity and the transcriptional activation of AP-1 target genes, including genes involved in inflammation and oxidative stress.

In *Drosophila*, the loss of *Pex19* enhanced mitochondrial β-oxidation and induced mitochondrial swelling [109]. The hepatocyte nuclear factor 4 (Hnf4) was identified as a key regulator of mitochondrial dysfunction in *Pex19* mutant flies [109]. Hnf4 is an important transcription factor that regulates mitochondrial fatty acid β-oxidation [146] and insulin secretion [147]. Mutations in *Pex19* activated Hnf4 and consequentially upregulated the expression of lipase 3 (*Lip3*) and lipolysis. The authors proposed that Hnf4-mediated lipotoxicity and accumulation of free fatty acids (FFAs) are the causes of mitochondrial damage (e.g., elevated ROS) in *Pex19* mutant flies [109]. The study also revealed that peroxisome deficiency not only triggers the accumulation of VLCFAs but also causes the depletion of shorter fatty acids. Consequently, lipolysis is elevated to produce high levels of FFAs, which hyper-activates Hnf4 signaling, further enhancing lipolysis and lipotoxicity (Figure 3).

### 5.2. Peroxisome-Mitochondrion Crosstalk

Peroxisomes and mitochondria share many common features. They metabolically cooperate in fatty acid β-oxidation and ROS metabolism [104]. The two organelles contain distinct sets of substrate-specific enzymes for fatty acid β-oxidation: VLCFAs are chain-shortened in the peroxisome, and then the medium- or short-chain fatty acids metabolites are guided to the mitochondria for further oxidation to CO_2_ and H_2_O as well as ATP production [6,148]. Moreover, the two organelles share multiple components of their division machinery including DRP1, FIS1, MFF, and ganglioside-induced differentiation-associated protein (GDAP1) [104].

The crosstalk between peroxisome and mitochondria is also supported by physical interaction through membrane contact sites. Recently, Shai et al. [149] reported two novel tethers (Fzo1 and Pex34) for peroxisome-mitochondrion contact in yeast. The peroxisomal membrane protein Pex34 potentially contributes to the transfer of β-oxidation products to mitochondria through the interaction with an unknown mitochondrial protein. Fzo1 on mitochondria may mediate mitochondria–peroxisome tethering either by interacting with the Fzo1 on the peroxisome or with another peroxisomal protein [149]. Several other tethers between peroxisomes and mitochondria have also been identified. For example, Pex11 on peroxisome to Mdm34 on mitochondria [150]; ABCD1 on peroxisome to unknown mitochondrial protein [151]; and, in mammals, ECI2 (enoyl-CoA delta isomerase 2, also known as ACBD2) on peroxisome to TOMM20 (translocase of outer mitochondrial membrane 20) on mitochondria [152] (Figure 2).

Mitochondria-derived vesicles (MDVs) have been identified as an intracellular transport route between mitochondria and peroxisomes [153]. Neuspiel et al. [153] showed that MDVs were formed independently of DRP1. Interestingly, MDVs containing MAPL, a mitochondrial small ubiquitin-like modifier E3 ligase, are targeted to fuse with a subset of peroxisomes. This study provided the first evidence of a direct relationship between the two organelles. Furthermore, the same group recently demonstrated that MDVs fused with ER-derived pre-peroxisomes to form mature functional peroxisomes [18].

As VLCFAs are catabolized into a range of chain-shortened fatty acids in peroxisomes and then fully oxidized in mitochondria, the metabolic intermediates produced from fatty acid β-oxidation could serve as metabolic signals in the communication between peroxisome and mitochondria. ABCD1 is a key transporter that delivers VLCFAs into the peroxisome for β-oxidation. The loss of *ABCD1* transporter in mice caused the accumulation of VLCFAs in the cytosol [75,154] accompanied by late-onset axonal degradation in the spinal cord and movement problems resembling the most common phenotype in X-ALD patients [155]. Lopez-Erauskin et al. [108] showed that impaired peroxisomal function due to the loss of *Abcd1* in the spinal cord resulted in severe mitochondrial abnormalities, such as the down-regulation of oxidative phosphorylation and mtDNA damage [108]. The authors also demonstrated that exposure of human fibroblasts of X-ALD patients to C26:0 caused mitochondrial dysfunction by increasing mitochondrial ROS production, mitochondrial oxidative damage to DNA and proteins, and eventually the disruption of oxidative phosphorylation [108]. However, Oezen et al. [156] reported that mitochondrial respiration in the skeletal muscle cells of *Abcd1*-knockout mice was normal, regardless of the accumulation of cytosolic VLCFAs. Moreover, mitochondrial morphology parameters such as size and structure in the muscle cells of X-ALD and control mice did not differ [156]. Taken together, mitochondrial susceptibility to accumulated VLCFAs may vary among tissues and cell types.

Plasmalogen is the most common form of ether lipids, and the initial steps of ether lipid biosynthesis take place in peroxisomes. Peroxisome-derived plasmalogen is responsible for mitochondrial dynamics and function [110]. The inhibition of plasmalogen synthesis by knocking down *GNPAT*, an enzyme responsible for the first step in plasmalogen biosynthesis, resulted in altered mitochondrial morphology and decreased mtDNA content. Similar to *Pex16* knockout, *GNPAT* knockdown resulted in elongated mitochondria, suggesting the potential disruption of mitochondrial fission [110]. Interestingly, high levels of plasmalogens were detected in the mitochondrial membrane [110], indicating that peroxisome-derived plasmalogen might be an important signal molecule that acts on the mitochondrial membrane to regulate mitochondrial membrane dynamics, especially the mitochondrial fission process (Figure 3).

### 5.3. Peroxisome-ER Crosstalk

Similar to peroxisome-mitochondrion crosstalk, the interaction between peroxisomes and ER was observed in the early years of peroxisome research [157,158]. Electron micrographs revealed that the two organelles are adjacent to each other, and peroxisomes are frequently seen wrapped around the ER membrane, implying a close connection between the two organelles [5,157,158]. Indeed, the membrane contact sites of peroxisome-ER have been recently discovered in mammalian cells [159,160]. Acyl-coenzyme A binding domain protein 5 (ACBD5) on the peroxisome membrane binds to the resident ER protein vesicle-associated membrane protein-associated protein B (VAPB). The interaction between the FFAT-like motif (two phenylalanines in an acidic tract) of ACBD5 and the MSP (major sperm protein) domain of the VAPB protein mediates the close association between the two organelles [161].

Peroxisome-ER communication plays an important role in maintaining lipid homeostasis, as the two organelles coordinate many lipid metabolic processes. For example, fatty acid oxidation in the peroxisome is strongly dependent on the crosstalk with the ER, since VLCFAs originate from the ER. Most of VLCFAs are not derived from dietary sources but synthesized from shorter-chain fatty acids through the fatty acid synthase (FAS) complex followed by the chain-elongation system localized in ER [162]. The physical interaction between peroxisome and ER is important for intracellular VLCFAs homeostasis since the genetic deficiency of ACBD5 results in the accumulation of VLCFAs [163]. In addition, peroxisome-ER interaction is also crucial for the biosynthesis of ether phospholipids, which is initiated in peroxisomes and completed in the ER. The loss of ACBD5 in fibroblasts resulted in a significant decrease in ether phospholipid biosynthesis, supporting the key roles of peroxisome-ER contact in the biosynthesis of ether phospholipids [164]. In addition, the disruption of peroxisome-ER contact inhibited peroxisomal membrane expansion and led to the formation of shorter peroxisomal membrane tubules and spherical organelles, suggesting a role for ACBD5-VAPB contact in peroxisomal membrane dynamics [160] (Figure 2).

Recently, Torres et al. [165] identified another peroxisome-ER tether mediated by the interaction between the peroxisomal fatty acid transporter ABCD3 and the ER-resident stress sensor ATF6α upon treatment with Ceapins, a new class of UPR inhibitor [165]. Treatment with Ceapins induced an ER-peroxisome association by tethering ATF6α to ABCD3, blocking its translocation to the Golgi apparatus [165,166,167] (Figure 2). As discussed in Section 4.5, recent studies show that peroxisomal dysfunction can activate ER stress response pathways, especially the PERK-eIF2α-ATF4 axis [98,121,122,123] (Figure 3). However, the underlying mechanisms for peroxisome-regulated ER stress response remain unknown. Does peroxisomal dysfunction activate PERK signaling through peroxisome-ER contact sites, similar to the interaction between ATF6α and ABCD3? Or is metabolic signaling (e.g., via VLCFAs) involved in the activation of PERK signaling? Future work is needed to address these possibilities.

### 5.4. Peroxisome-Lysosome Crosstalk

Lysosomes play an important role in the degradation of biomolecules, including unwanted fatty acids in cells. Several lines of evidence have demonstrated a strong link between peroxisomes and lysosomes. For instance, Chu et al. [103] demonstrated the physical interaction between lysosomes and peroxisomes mediated through the interaction between lysosomal synaptotagmin VII (Syt7) and the peroxisome membrane-bound phosphatidylinositol 4,5-bisphosphate (PI (4,5) P_2_) (Figure 2). These lysosome-peroxisome membrane contacts are essential for the transport of cholesterol from lysosomes to peroxisomes. Kleinecke et al. [168] provided in vivo evidence for functional interactions between peroxisomes and lysosomes. The authors showed that peroxisomal dysfunction associated with Schwann cell-specific *Pex5* knockout caused the impairment of lysosomes in peripheral nerves, resulting in the perturbation of normal ganglioside turnover. This led to the abnormal distribution of potassium ion channels, possibly disturbing the proper signal transmission in the peripheral nerves of Schwann cell-specific *Pex5* knockout mice [168].

Peroxisomes have also been shown to interact with the autophagy-lysosome system. He et al. [139] showed that hepatocyte-specific *Acox1* knockout mice were protected from high-fat diet-induced liver steatosis and fatty liver diseases. The study further showed that the loss of *Acox1* led to decreased acetyl-CoA levels and the acetylation of Raptor, a key subunit of mTORC1, which likely induced autophagy through Unc-51-like autophagy-activating kinase 1 (ULK1). These findings suggest that peroxisomal acetyl-CoA might act as an important signaling metabolite to regulate mTORC1, lipophagy, and lipid homeostasis [139] (Figure 3).

## 6. Peroxisomal Dysfunction in Aging and Aging-Related Diseases

Several lines of evidence suggest that peroxisomal dysfunction is an underappreciated cause of aging [11,12,13,14]. Peroxisomal protein import is known to be compromised in aged tissues [11,12,13,14], and almost all peroxisomal proteins are downregulated during aging [8,13]. In senescent human fibroblasts, catalase import was significantly reduced, leading to the accumulation of H_2_O_2_ and the further disruption of peroxisome import [11]. Conversely, the overexpression of *Pex5* has been shown to restore peroxisomal import function and preserve cardiac health in aged oenocytes [14]. Importantly, Sebastiani et al. [169] studied the proteomic signatures of centenarians and identified that proteins encoding components of peroxisomes were significantly downregulated in centenarians’ serum compared with younger individuals. In addition, the bile acid metabolism pathway and cholesterol homeostasis pathway were also downregulated in centenarians. These studies provide strong evidence for a role of impaired peroxisomal homeostasis in aging.

As peroxisomal activities decline with age [170], it has been suggested that peroxisomal dysfunctions might be associated with the pathogenesis of age-related neurodegenerative disease, including Alzheimer’s disease [171], Parkinson’s disease [172], and amyotrophic lateral sclerosis [173]), as well as age-related metabolic disease, such as cardiovascular disease [174], obesity [76], diabetes [175], and nonalcoholic liver disease [176]. These age-related pathologies are likely due to increased oxidative stress [177,178] and decreased plasmalogen synthesis [179]. In this section, we summarize the current knowledge about the role of peroxisomes in several age-related diseases.

Alzheimer’s disease (AD) is the most common neurodegenerative disease in the elderly. It is characterized by the progressive loss of synapses and neurons especially in the frontal cortex and hippocampus accompanied by significant memory loss and cognitive impairment [180,181]. The pathological hallmarks of AD are the accumulation of the extracellular beta-amyloid (Aβ) plaques and intracellular fibrillary deposits of hyperphosphorylated tau protein (neurofibrillary tangles). Oxidative stress is thought to be a primary culprit in AD pathogenesis [182,183]. Accordingly, the hippocampus of AD mice was found to exhibit high levels of markers of lipid peroxidation and DNA/RNA oxidation [182]. Several lines of evidence suggest a link between peroxisomal dysfunction and AD pathogenesis. The accumulation of VLCFAs and decreased plasmalogen levels were observed in the cortical regions of AD patients [184]. Also, the levels of DHA in the brain and liver tissue of AD patients were reduced compared to those in control individuals [185]. Impaired peroxisomal function might also contribute to alterations of peroxisomal metabolites in AD pathology. Shi et al. [186] showed that the inhibition of peroxisomal β-oxidation increased the level of VLCFAs and Aβ contents in rat cerebral cortex. On the other hand, treatment with peroxisome proliferators attenuated AD-related pathology and improved spatial memory in transgenic mice models of AD [187]. Although peroxisomal dysfunction is associated with AD pathogenesis, whether decreased peroxisomal activity is the primary cause, bystander, or consequence in AD pathology remains to be determined [188].

Cardiovascular diseases (CVDs) are the major cause of death in elderly populations [189], and the prevalence of CVDs has been shown to increase with age [190]. Aging plays a critical role in the impairment of the cardiovascular system and increases the risk of CVDs [191]. Peroxisomal dysfunction has been implicated in CVDs. For instance, cardiac-specific overexpression of catalase, predominantly located in peroxisomes [192], was found to prevent the progression to overt heart failure [174]. In addition, catalase activity is significantly decreased in failing myocardium [193]. Refsum’s disease is a peroxisomal disorder caused by the impaired α-oxidation of branched-chain fatty acids, and patients with Refsum’s disease develop cardiac arrhythmia and heart failure later in life [194,195,196]. We recently demonstrated that peroxisomal dysfunction in *Drosophila* liver induced cardiac arrhythmia through the production of inflammatory cytokine *upd3/IL-6* [14]. We showed that the production of *upd3/IL-6* activated the cardiac JAK-STAT signaling pathway and induced cardiac arrhythmia. Our studies provide direct evidence connecting peroxisomal dysfunction to systemic inflammation and age-related cardiomyopathy [14].

Aging is accompanied by changes in the distribution and composition of adipose tissue, particularly an increase in fat deposition within the white adipose tissue (abdominal obesity), which is a major contributor to insulin resistance and metabolic syndrome [197,198,199]. As life expectancy increases, the prevalence of obesity has also risen steadily among the elderly [199]. A recent study demonstrated that peroxisomes play an important role in adipose dysfunction associated with obesity [76]. The authors found that obese mice exhibited downregulation of peroxisomal genes in white adipose tissue, and the knockdown of *Pex5* increased ROS levels and inflammation in adipocytes. Accordingly, catalase knockout mice also showed accelerated obesity compared to control mice. These phenotypes were attenuated by improving peroxisomal biogenesis through treatment with the PPARα agonist fenofibrate [76]. Additionally, catalase-deficient patients frequently show diabetic pathology, presumably due to the accumulation of oxidative damage in pancreatic β-cells [200].

## 7. Concluding Remark

Aside from their role in metabolic regulation, peroxisomes have emerged as a regulatory hub for maintaining cellular and metabolic homeostasis. The perturbation of peroxisomal function has been linked to tissue functional decline and age-related diseases. Recent in vitro and in vivo studies have revealed a close connection between peroxisomes and other subcellular organelles. These inter-organelle communications, often through organelle membrane contact sites, are essential for maintaining normal peroxisome function, as well as the homeostasis of other organelles. It is evident that a homeostatic signaling pathway exists in eukaryotic cells to cope with malfunctioning peroxisomes and to protect cells from damages, as various cellular and metabolic changes are reported upon peroxisomal dysfunction. These findings open a new avenue for research on the “peroxisomal stress response pathway”.

Cellular stress responses are the mechanisms by which cells adapt to stressful conditions in order to restore normal cellular function [201]. Although peroxisome disfunction significantly contributes to the loss of cellular homeostasis and the activation of many cellular signaling pathways (e.g., ER stress), the cellular mechanisms by which the cell senses malfunctioning peroxisomes and activates the appropriate cytoprotective responses are still largely unknown. To fully characterize the peroxisomal stress response pathway, further research is needed to identify novel signaling molecules (including signaling metabolites) that relay peroxisomal stress to other parts of the cell and modulate the functions of other subcellular organelles. Advanced imaging techniques, including super-resolution live-cell imaging, will greatly facilitate the analysis of organelle membrane contact sites and membrane dynamics. Targeted lipidomics analysis of putative signaling metabolites will reveal new metabolic signaling pathways that mediate peroxisome-organelle crosstalk and metabolic homeostasis. Lastly, since peroxisomal dysfunction has been implicated in aging and aging-related diseases, such as Alzheimer’s disease, cardiovascular diseases, and diabetes, understanding peroxisomal stress responses will shed light on the potential of targeting the peroxisome as a therapeutic approach for aging-related diseases.
